# Supplementary material for: Online Health Information-Seeking Behaviours and eHealth Literacy among First-Generation Chinese Immigrants
Source: Int J Environ Res Public Health. 2023 Feb 16;20(4):3474. doi: 10.3390/ijerph20043474 (PMC9965195; doi:10.3390/ijerph20043474)
Supplement: Supplementary file 1 [file ijerph-20-03474-s001.zip › ijerph-2088357-supplementary.pdf]

## Correlations

| | | Age | Years lived in Au | English proficiency | Education levels | No of chronic conditions | Health levels | No of technology devices used | eHealth literacy scores |
|---|---|---|---|---|---|---|---|---|---|
| Age | Pearson Correlation | 1 | .130* | .344** | -.439** | .519** | .570** | -.558** | -.465** |
| | Sig. (2-tailed) | | .014 | <.001 | <.001 | <.001 | <.001 | <.001 | <.001 |
| | N | 362 | 356 | 356 | 360 | 361 | 360 | 362 | 362 |
| Years lived in Au | Pearson Correlation | .130* | 1 | -.077 | -.079 | .033 | .047 | -.010 | -.043 |
| | Sig. (2-tailed) | .014 | | .148 | .139 | .532 | .375 | .855 | .422 |
| | N | 356 | 356 | 350 | 354 | 355 | 354 | 356 | 356 |
| English proficiency (excellent to poor) | Pearson Correlation | .344** | -.077 | 1 | -.166** | .165** | .266** | -.205** | -.195** |
| | Sig. (2-tailed) | <.001 | .148 | | .002 | .002 | <.001 | <.001 | <.001 |
| | N | 356 | 350 | 356 | 354 | 355 | 354 | 356 | 356 |
| Education levels (primary/secondary/ university) | Pearson Correlation | -.439** | -.079 | -.166** | 1 | -.287** | -.396** | .479** | .447** |
| | Sig. (2-tailed) | <.001 | .139 | .002 | | <.001 | <.001 | <.001 | <.001 |
| | N | 360 | 354 | 354 | 360 | 359 | 358 | 360 | 360 |
| No of chronic conditions | Pearson Correlation | .519** | .033 | .165** | -.287** | 1 | .507** | -.272** | -.304** |
| | Sig. (2-tailed) | <.001 | .532 | .002 | <.001 | | <.001 | <.001 | <.001 |
| | N | 361 | 355 | 355 | 359 | 361 | 359 | 361 | 361 |
| Health levels (excellent to poor) | Pearson Correlation | .570** | .047 | .266** | -.396** | .507** | 1 | -.434** | -.423** |
| | Sig. (2-tailed) | <.001 | .375 | <.001 | <.001 | <.001 | | <.001 | <.001 |
| | N | 360 | 354 | 354 | 358 | 359 | 360 | 360 | 360 |
| No of technology devices used | Pearson Correlation | -.558** | -.010 | -.205** | .479** | -.272** | -.434** | 1 | .457** |
| | Sig. (2-tailed) | <.001 | .855 | <.001 | <.001 | <.001 | <.001 | | <.001 |
| | N | 362 | 356 | 356 | 360 | 361 | 360 | 362 | 362 |
| eHealth literacy scores | Pearson Correlation | -.465** | -.043 | -.195** | .447** | -.304** | -.423** | .457** | 1 |
| | Sig. (2-tailed) | <.001 | .422 | <.001 | <.001 | <.001 | <.001 | <.001 | |
| | N | 362 | 356 | 356 | 360 | 361 | 360 | 362 | 362 |

*. Correlation is significant at the 0.05 level (2-tailed).

**. Correlation is significant at the 0.01 level (2-tailed).
